# Nanoarchitectonics in Materials Science, Second Edition

**DOI:** 10.3390/ma19040820

**Published:** 2026-02-21

**Authors:** Katsuhiko Ariga, Rawil Fakhrullin

**Affiliations:** 1 Research Center for Materials Nanoarchitectonics (MANA), National Institute for Materials Science (NIMS), 1-1 Namiki, Tsukuba 305-0044, Ibaraki, Japan; 2 Graduate School of Frontier Sciences, The University of Tokyo, 5-1-5 Kashiwanoha, Kashiwa 277-8561, Chiba, Japan; 3 Institute of Fundamental Medicine and Biology, Kazan Federal University, Kreml uramı 18, Kazan 42000, Republic of Tatarstan, Russia; 4 Biological Institute, Tomsk State University, 36 Lenin Ave., Tomsk 634050, Russia

There are many social needs that nanoarchitectonics, as an emerging technology, can meet, such as converting and storing energy [1,2,3], cleaning the environment, and sensing toxic substances [4,5,6]. It can also be used in various biomedical applications [7,8,9]. One important way that science and technology can meet these demands is by developing new materials that have special predetermined functions. Human progress has been driven by the constant evolution of materials science. For example, there have been advances in inorganic chemistry [10,11,12], which is the study of metals, ceramics, and various inorganic materials. Research is also underway in organic chemistry [13,14,15]; polymer chemistry [16,17,18], which concerns the development of organic and polymeric materials; supramolecular chemistry [19,20,21], which concerns their assemblies; coordination chemistry [22,23,24], which is associated with inorganic materials; and biochemistry [25,26,27], which bridges the biological world and materials science. These scientific advances have made it possible to create a huge number of different materials, and these new materials have made it possible to create devices and technologies that can meet a variety of needs and improve human lives.

As we have developed this Special Issue, it has become clear that controlling materials and how they are structured can enable them to work more efficiently and change their characteristics. In other words, we now understand that it is not enough to just produce functional materials; we also need to control their intrinsic nanostructure [28,29]. To make materials that are better than their regular counterparts, we need to improve them and control how they are shaped and assembled. Thus, as well as the usual ways of making products and creating materials, we need to use the latest technology to control structures at the atomic/molecular and nanometer scales [30,31]. Specifically, there is growing demand for materials that use nanotechnology.

As is universally agreed, nanotechnology is very important in the development of materials, including very small structures. However, nanotechnology is not a specialized academic subject focused on making materials; its main focus is on understanding new nanoscale events and the physical principles behind them [32,33]. Making useful materials from very small building blocks requires work in other areas, like supramolecular chemistry, materials processing, and biotechnology. Thus, functional materials at the nanometer scale should be created using a new approach that combines the different research areas mentioned above as well as nanotechnology. This idea is called “nanoarchitectonics” [34]. We need to develop an approach that brings together “normal” science and technology with the study of miniscule (in other words, “nano”) things. Nanoarchitectonics is responsible for achieving this.

With nanotechnology, we can arrange atoms precisely or fix a single molecule. However, even if we could make a device made from just one molecule, it would be useless on its own. These elements need to be joined together to create a system that has new and exciting features. In reality, nanotechnology can already facilitate the production of very small structures, but it does not possess the ability to join them together so they work properly. Although there have been attempts to create nanostructures using supramolecular chemistry and then organize them to create higher-order structures [35,36], this is still a very new field and, so far, has been fairly unsuccessful. However, this is exactly why it remains a field with great potential. Nanotechnology will probably only be useful to a limited extent until we have developed a technological framework for organizing and constructing nanostructures through nanoarchitectonics.

Making working systems using nanotechnology will probably require a method based on conventional microfabrication technology. This means first developing a plan for a perfect system, and then building a structure according to it. Biological systems are a superior kind of functional systems. They are designed to function while accepting the effects of Brownian motion and thermal fluctuations. While the brain is often described as a computer, in the first approximation, it uses only ionic currents, not electronic ones. Living entities have evolved considerably over time, but the way they work is not at all like modern nanotechnology, which uses very small electronic circuits. Perhaps in the future, computers and other machines will be made in a similar way to living creatures. However, this might not go to plan and there may be some unexpected problems. However, these problems can also be exploited to create new and exciting things. This is not considered part of normal nanotechnology; instead, it must be developed as a new way of researching within the field of nanoarchitectonics.

One of the most important goals of nanoarchitectonics is to create highly functional structures, such as those found in living organisms, from basic units such as functional molecules [37]. Many biochemical systems are very efficient and specific because they have hierarchical and asymmetric structures. To achieve this, these structures enable relays and combinations of processes [38,39,40]. These hierarchical structures usually cannot be made using normal self-assembly in equilibrium; instead, they are made by taking a similar approach to the way that energy is used in biological systems, where components are assembled in non-equilibrium. It is important to use non-equilibrium forces in the process of making nanoarchitectonics structures. For example, adding artificial structures to a mixture in stages, using methods like the Langmuir–Blodgett method [41,42] or layer-by-layer adsorption [43,44], can enable the creation of structures with a layered and uneven pattern. Nanoarchitectonics, which combines these processes in a balanced way, could become a universal method of assembling highly functional systems, like those found in living organisms.

According to these historical, scientific, and technological backgrounds, the Special Issue entitled “Nanoarchitectonics in Materials Science” collected several relevant research papers on functional materials inspired by the nanoarchitectonics concept. This Special Issue was mainly based on a collection of papers published in *Materials* from late 2022 to early 2024. However, rapid progress has continued in materials sciences, as seen in various research papers from 2024 and 2025. These research trends heavily depend on material innovations with structural control at the nanostructure level and mesoscopic scale in many research fields. Continuous development in various key materials has been made in perovskite photovoltaics [45,46], organic semiconductors [47,48], mesoporous materials [49,50], metal–organic frameworks [51,52], and quantum materials [53,54]. Basic strategies including molecular synthesis [55,56], material production [57,58], polymer technology [59,60], self-assembly and self-organization [61,62], and host–guest systems [63,64] remain important in the production of nanostructure-based functional materials that can be used in many useful applications in the energy [65,66], environmental [67,68], and biomedical fields [69,70]; catalysis [71,72]; and material separation [73,74]. In addition to these well-recognized fields, new concepts in material controls such as single-molecule chemistry [75] and dynamic covalent chemistry [76] have been recognized, along with novel evaluation techniques including atomic-resolution electron microscopy [77] and quantum beam analysis [78]. All of this progress promotes rapid improvements in nanostructure-based materials science, leading to fruitful developments in nanoarchitectonics. Based on the rapid developments seen in the past few years, “Nanoarchitectonics in Materials Science, Second Edition” was launched, and the research papers collected in it are summarized below.

Three review papers highlight the importance of nanoarchitectonics approaches, from basic to advanced applications. Nanoarchitectonics is defined as a fascinating frontier and a method for many processes in materials science [79]. Yang and Skirtach reviewed the roles of nanoarchitectonics approaches for sustainable food packaging as representative practical usages [80]. As another consideration of practical applications, Han, Jia, and co-workers, in their review article, discussed the applications of hydrogel-based triboelectric nanogenerators in intelligent sports [81]. Contributions to particular materials and specific applications are also included in this Special Issue. Cadenas-Pliego, Pérez-Alvarez, and co-workers discuss the surface modification nanoarchitectonics of TiO_2_ and ZrO_2_ nanoparticles using lactic acid and stearic acid for enhancing their antibacterial activity (Contribution 1). In the article by Yao and co-workers, they numerically evaluated nanoporous material liquid systems for use in mitigating blast effects on fiber composite circular structures (Contribution 2). Firmino, Menezes, and co-workers report the fabrication of nickel ferrite fibers using the solution blow spinning method, which was used for the adsorptive removal of anionic Congo red dye (Contribution 3). Ma et al. prepared S and N co-doped low-dimensional C/C nanocomposites with polymer and graphene oxide nanoribbons using one-pot carbonization through dimensional-interface and phase-interface tailoring of nanocomposites (Contribution 4).

However, this is only a fraction of what nanoarchitectonics can offer. Nanoarchitectonics is the principle of creating materials by assembling atoms and molecules, which could be a good method of making all materials. In the same way that the Theory of Everything in physics is the ultimate explanation of how the universe works [82], nanoarchitectonics could be referred to as the Method for Everything in materials science [83]. Nanoarchitectonics is being used more and more, regardless of the material, function, or application. It is used in fields that focus on the basic building blocks of matter, such as how matter is created, how structures are controlled, the fundamental physical properties of matter, and academic biochemistry. It is also used in applied fields, such as catalysis, sensors, devices, the environment, energy, and biomedicine.
